# A coupled hidden Markov model for disease interactions

**DOI:** 10.1111/rssc.12015

**Published:** 2013-05-06

**Authors:** Chris Sherlock, Tatiana Xifara, Sandra Telfer, Mike Begon

**Affiliations:** Lancaster UniversityUK; University of AberdeenUK; University of LiverpoolUK

**Keywords:** Adaptive Markov chain Monte Carlo sampling, Forward–backward algorithm, Gibbs sampler, Hidden Markov models, Zoonosis

## Abstract

To investigate interactions between parasite species in a host, a population of field voles was studied longitudinally, with presence or absence of six different parasites measured repeatedly. Although trapping sessions were regular, a different set of voles was caught at each session, leading to incomplete profiles for all subjects. We use a discrete time hidden Markov model for each disease with transition probabilities dependent on covariates via a set of logistic regressions. For each disease the hidden states for each of the other diseases at a given time point form part of the covariate set for the Markov transition probabilities from that time point. This allows us to gauge the influence of each parasite species on the transition probabilities for each of the other parasite species. Inference is performed via a Gibbs sampler, which cycles through each of the diseases, first using an adaptive Metropolis–Hastings step to sample from the conditional posterior of the covariate parameters for that particular disease given the hidden states for all other diseases and then sampling from the hidden states for that disease given the parameters. We find evidence for interactions between several pairs of parasites and of an acquired immune response for two of the parasites.

## 1. Introduction

### 1.1. Motivating problem

In natural populations, animals are likely to be infected by a variety of pathogens, either simultaneously or successively. Interactions between these pathogens, which can be synergistic or antagonistic, can affect infection biology (e.g. the intensity of one or both infections), or host susceptibility to infection, or may impact on the host’s morbidity or/and mortality. However, the biological processes that are involved are often too complex to allow clear-cut predictions regarding the outcome of such interactions. To explore potential interactions, a longitudinal study was undertaken by recording the sequences of infection events for different parasites in four spatially distinct populations of field voles (*Microtus agrestis*). The data are records of six pathogens: three species of *Bartonella* bacteria (*B. taylorii*, *B. grahamii* and *B. doshiae*), cowpox virus, the bacterium *Anaplasma phagocylefthilum* and the protozoan *Babesia microti*. Aside from their intrinsic interest as a community of pathogens, *Bartonella,Anaplasma,Babesia* and cowpox virus infections may also be zoonotic: capable of being transmitted from animals to humans and causing disease.

As in most capture–mark recapture studies, a different set of voles was caught at each session leading to incomplete profiles for all subjects. The data set therefore contains many missing observations; for example a profile for a given vole and a given disease from the first to last observation times for that vole might be NPxxPNxP, where x, N and P respectively indicate a missing observation, a negative response and a positive response. Inference on incomplete data in longitudinal and capture–recapture studies is a major problem; for examples see Daniels and Hogan (2008) and Pradel (2005). Previous analyses of our and related data sets (see Telfer *et al.* (2010) and Begon *et al.* (2009)) have examined all pairs of observations for a given vole that occurred exactly 1 lunar month apart and for which the first of the two observations was an N. The influence of each covariate on the probability of contracting a disease is then ascertained through logistic regression. In this paper we offer a more realistic model and a more powerful analysis methodology for investigating the effects of previous infections for each disease on the other diseases. We use a hidden Markov model (HMM) for each disease (Section 2.1) and perform inference via a Gibbs sampler; this allows us to use all of the data set and to infer covariate effects on a given disease, even when these covariates are the (potentially missing or hidden) states of the other five diseases.

### 1.2. Data

We analyse data collected between March 2005 and March 2007 from field voles in Kielder Forest, which is a man-made forest on the English–Scottish border. The voles were trapped at four grassy clear-cut sites within the forest, with each site at least 3.5 km from the nearest neighbouring site. Individuals were trapped within a 0.3 ha live trapping grid comprising 100 traps set at 5 m intervals, with trapping taking place every 28 days from March to November, and every 56 days from November to March. Begon *et al.* (2009) have provided further details of the study area and the trapping design.

Captured voles were marked with a unique identifying passive transponder tag to be recognized in later captures. At each capture, a 20–30-*μ*l blood sample was taken for pathogen diagnostic tests. Polymerase chain reaction assays were used to test directly for evidence of infection with *Anaplasma phagocylefthilum*, *Babesia microti* and the three *Bartonella* species (see Courtney *et al.* (2004), Bown *et al.* (2008) and Telfer *et al.* (2008)). Antibodies to cowpox virus were detected by immunofluorescence assay (see Chantrey *et al.* (1999)). A brief description of the observed and derived variables is given in Table 1.

**Table 1:** Description of variables in the data set and their possible outcomes

*Variable*	*Description*
Tag	Unique number that identifies each vole
Site	Identifier for the capture site (four-level factor)
Sex	Male or female
Lm	Capture time point in whole lunar months (1–27; integer)
Weight	Weight in grams rounded to the nearest 0.5 g
Sin	sin (2*π* Lm/13)
Cos	cos (2*π* Lm/13)
Tay	*B. taylorii*, N (negative) or P (positive)
Grah	*B. grahamii*, N (negative) or P (positive)
Dosh	*B. doshiae*, N (negative) or P (positive)
Cow	Cowpox, N (negative) or P (positive)
Ana	*Anaplasma*, N (negative) or P (positive)
Bab	*Babesia*, N (negative) or P (positive)

After some processing (which is described in detail in Xifara (2012)) our data set contains 4344 captures of 1841 voles. Only voles that have been caught at least twice are directly informative about transition probabilities (see Section 2.1), although voles that have been captured only once still contribute to inference for the initial distribution of each hidden Markov chain (see Section 3.1.1).

The data set contains a substantial fraction of missing data: almost half of the voles are not captured at every lunar month between the first and last times that they were observed. Thus, even for many of the voles that were observed at least twice, not all of the covariates are available, either because the vole was not caught in a given lunar month, or sometimes because the vole was caught but a given variable was not ascertained. Table 2 shows the frequency of missing values derived from the first cause. The number of additional missing values, where it was not possible to ascertain the status of a particular disease, despite the vole being captured, is given in Table 3, which also shows the frequency of positive (P) and negative (N) records for each disease.

**Table 2:** Frequency of missing values per vole as a function of the number of lunar months from the first capture time to the last capture time

*Lunar months from first to last capture*	*Frequencies for the following numbers of values missing*:				
	*0*	*1*	*2*	*3*	≥ *4*
0	832	—	—	—	—
1	275	—	—	—	—
2	132	74	—	—	—
3	75	55	15	—	—
4	30	49	33	9	—
5	21	24	34	5	3
6	7	7	33	15	2
7	1	4	25	16	9
<7	0	2	27	12	15

**Table 3:** Summary information for the six diseases; number of missing values despite the vole being captured and numbers of negative N and positive P responses

*Disease*	*Number of additional*	*Number*	*Number*
	*missing values*	*of N*	*of P*
*B. doshiae*	46	3583	715
*B. grahamii*	44	3468	832
*B. taylorii*	32	3139	1173
*Babesia*	0	2354	1990
Cowpox	85	1408	2851
*Anaplasma*	6	4107	231

### 1.3. Statistical challenges

We aim to investigate potential interactions between the six pathogens of the study. In particular, for each disease *d*, we wish to evaluate the way in which the presence or absence of each of the other diseases (and perhaps further information such as whether or not any infection is in its first month) affects the probability that vole contracts *d*. Additionally where applicable we are interested in how other diseases affect the probability of recovery from *d*.

We could model each disease as a two-state discrete time Markov chain, where state 1 corresponds to no disease and state 2 to presence of disease; however, this two-state model imposes a very specific structure. For example the length of any infection is geometrically distributed; however, it might be that the probability of remaining infected when a disease is in its first month (an acute phase) is different from that in subsequent months (chronic phase). It has also been found (e.g. Telfer *et al.* (2010)) that acute and chronic phases of a disease 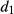 can have different effects on the probability that a vole contracts disease 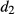. A two-state semi-Markov model (see, for example, Guédon (2003)) could account for the first effect, at the expense of extra complexity, but not the second. To represent both the dynamics and the influence of each disease with minimal extra complexity adequately, therefore, in this analysis the dynamics of all except one of the diseases is modelled as a Markov chain with more than two states. Section 2.1 details the model for each disease.

Only knowledge of the presence or absence of the disease is available to us. In general, this equates to knowledge of a subset of the state space in which the true state must lie, but not to the exact state of the chain. For example, for all except one disease, states 2 and 3 both correspond to the presence of the disease. In disease modelling, HMMs arise when the Markov model for disease progression has several stages, or states, but these are not directly observed (e.g. Guihenneuc-Jouyaux *et al.* (2000) and Chadeau-Hyam *et al.* (2010)). Often the relationship between the state of the Markov chain and the observation is stochastic, although in our case no stochasticity is involved, but the state of the Markov chain is nonetheless hidden. Furthermore, observations are only available to us when the vole has been captured. The forward–backward algorithm (see Section 2.3) can be applied to any discrete time HMM with a finite state space and addresses both of these issues.

We consider *D* = 6 diseases, and hence six interacting (or coupled) HMMs. It is possible to consider the coupled Markov chains for each disease together as a single Markov chain on an extended state space. In this case the likelihood function is straightforward to evaluate by using the forward–backward algorithm (see for example Zucchini and MacDonald (2009)) and a Bayesian analysis can then be performed by using Markov chain Monte Carlo (MCMC) methods. In our particular scenario the state spaces have size 4, 4, 4, 3, 3 and 2, which would lead to an extended state space of size 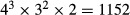. Since the forward–backward algorithm applied to an HMM with *n* states takes 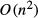 operations, a naive implementation of the algorithm applied to the extended state space would have a complexity of 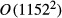 compared with a complexity of 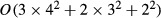 for six coupled chains; equivalently, 100000 iterations of an algorithm which deals with each chain separately would take approximately the same central processor unit time as five or six iterations of the single-chain algorithm. In our specific scenario, but certainly not in generality, some of the transition probabilities in each individual chain are 0, and (in our scenario) only 32 768 elements of the extended transition matrix would be non-zero. The use of sparse matrix routines could therefore reduce the efficiency ratio to approximately 468. Such a reduction in computational efficiency would only be justified if the fraction of missing data were very close to 1 so that the mixing of our Gibbs sampler would be extremely slow.

Pradel (2005) analysed capture–recapture data by using an HMM, and incorporation of covariate information within this framework via an appropriate link function is straightforward (see Lachish *et al.* (2011) and Zucchini and MacDonald (2009) (section 8.5.2)). However, the methodology does not allow the use of multiple HMMs nor, therefore, can it use the state of each HMM as a covariate for the other HMMs. We require six HMMs (one for each disease) and we wish to use covariate information such as the time of year and weight of the vole. Furthermore we wish the covariate set for each disease to include the states of the HMMs for the other diseases. For each disease *d*, we shall represent the probability of each possible state change through a logistic regression. However, some of the covariates, the states of the other HMMs, are unknown. Our solution is a Gibbs sampler which employs the forward–backward algorithm and adaptive random-walk Metropolis steps to sample from the true posterior distribution of all of the HMMs and the covariate parameters jointly.

### 1.4. Outline

The remainder of this paper is organized as follows. Section 2 describes the model which was used for each disease, gives its likelihood function and outlines the imputation of missing weight values and the other fixed covariate values. The MCMC algorithm is described in Section 3 and we present our results, including the sensitivity study, in Section 4. The paper concludes with a discussion.

The data that are analysed in the paper can be obtained from http://www.blackwellpublishing.com/rss

## 2. Modelling the hidden and missing data

### 2.1. Hidden Markov models and notation

HMMs are used when observations are influenced by a Markov process but the state of the Markov process itself cannot be determined exactly from the observations. Usually the relationship between the Markov process and the observation process is stochastic, but (as in our application) this need not be so. For various examples and applications of HMMs see, for example, Zucchini and MacDonald (2009). Purely to simplify our subscript notation we consider each vole to have been first observed at a local (to the vole) time of 1 and last observed at (local) time *T*. For disease *d* (*d*=1,…,*D*), let the state space for the Markov chain be 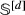 and the state space for the observation process be 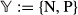. For a given vole and for disease *d*, the unobserved Markov chain and the observations are respectively 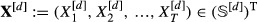 and 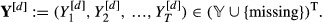


Note that 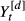 is conditionally independent of 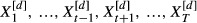 given 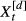. The observed process 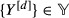 is related to the state of the hidden process 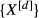 by a likelihood vector 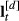, which has elements 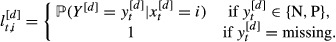


This vector is defined for each disease in Section 2.5.

We take the discrete time interval of each Markov chain to be 1 lunar month. Since trapping sessions in winter took place every 2 lunar months (see Section 1.2) this inevitably leads to missing observations for any vole caught several times over the winter, even if it is caught at every trapping session. For each unknown transition probability (see Section 2.5) we have a logistic regression model; for example the probability 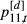 that a given vole will be in state 1 (disease *d* absent) at time *t*+1 given that it is in state 1 at time *t* is given by 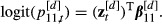


Here 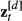 is the vector of covariates at time *t*, which for all models includes the states of the other diseases at time *t*, 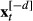, as well as a deterministic covariate vector 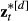. This deterministic vector was chosen via forward fitting of logistic regression models that were very similar to those of Telfer *et al.* (2010) and Begon *et al.* (2009) (see Section 1.1). However, whereas Telfer *et al.* (2010) and Begon *et al.* (2009) allowed both the current covariates and covariates 1 lunar month into the future to influence the response 1 lunar month into the future, we allow only the current covariates to influence the future response; further details are available in Xifara (2012). For all diseases the deterministic vector consists of a time trend (lunar month Lm as a continuous covariate), a seasonal cycle in the form of sin and cos, and sex, weight and site. The covariate vector for cowpox also includes a different trend with lunar month for each site, and for all other diseases it allows for a different seasonal cycle for each sex (see Table 1 for detailed covariate descriptions).

**Figure 1:**
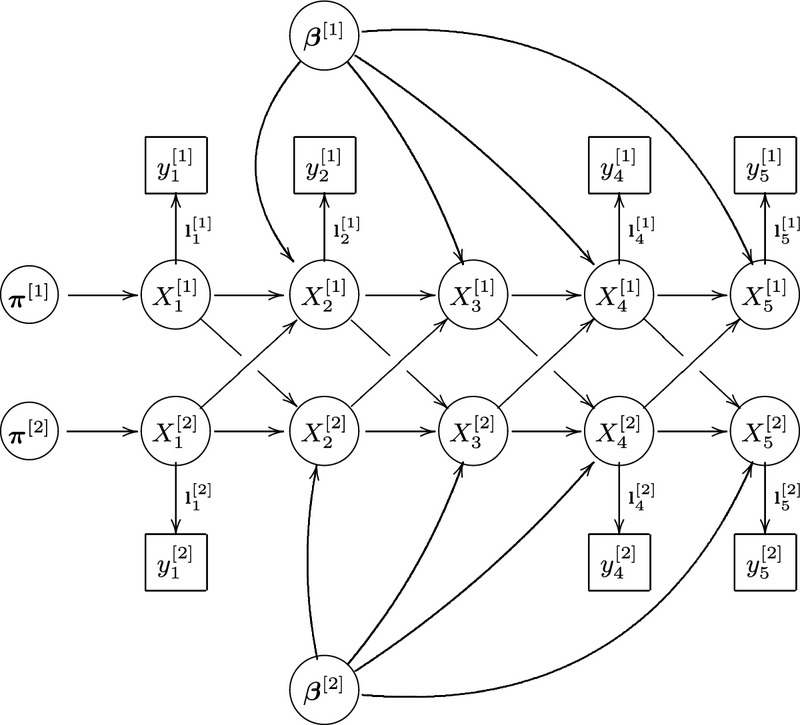
Directed graph of a realization of two parallel HMMs where 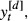 *t*=1,…,5, where present, are the observed values of disease *d* (*d*=1,2) and 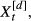 *t*=1,…,5, are the states of the hidden Markov chain for *d*, arising from (unknown) initial distribution 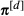: the nodes from 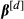 reflect dependence of the transition matrix on the (unknown) covariate parameters; to simplify the presentation and to focus on the coupled HMM we omit the deterministic covariate vectors 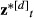.

We denote the transition probability matrix from time *t* to *t*+1 for disease *d* by 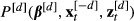, i.e. 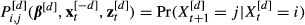, and let the initial distribution for the hid den chain be 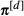. Fig. 1 depicts a simplification of our scenario, where there are just two diseases. Note that the states of all chains at time *t*+1, 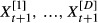, are independent conditional on the states of all chains at time *t*.

### 2.2. Likelihood function

We now provide full detail of the likelihood for a given vole. The likelihood for the data is simply the product of these likelihoods over all 1841 voles. Let 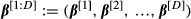, 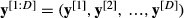 and 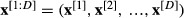. The conditional independence structure leads to a complete-data likelihood of 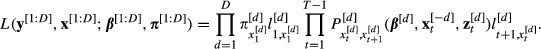


The observed data likelihood for the vole is then 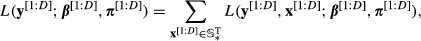
(1) where 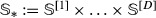. For a single chain the summation over the hidden states can be written as a matrix product; this simplification is not possible for coupled chains as the transition matrix for each disease depends on the state of each of the other diseases. Our Bayesian analysis requires prior distributions for 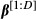 and 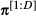, which are detailed in Section 3.1. The product of the observed data likelihoods over all voles multiplied by the prior distribution for 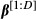 and 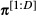 gives, up to a constant of proportionality, the joint posterior for 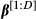, 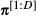 and 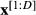.

### 2.3. The forward–backward algorithm

The forward–backward algorithm that was developed by Baum *et al.* (1970) (see also Zucchini and MacDonald (2009), Scott (2002), Rabiner (1989) and Chib (1996), for example) may be applied to any discrete time HMM with a finite state space and provides us with two useful tools. The *forward recursion* is a computationally efficient algorithm for calculating the likelihood of the observed data, whereas the *backward recursion* provides us with the distribution of each hidden state 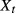, given the state at the next time point, 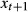, and all of the observations. Both will form part of the Gibbs sampling scheme that will be described in detail in Section 3.2.

### 2.4. Other missing covariates

As mentioned in Section 1.3, for many voles not all of the covariates are available. For a given vole, the covariates sex, site and Lm clearly carry over to the missing records. The unobserved disease states will be treated dynamically and will be sampled from the conditional distribution as part of the Gibbs sampling scheme (see Section 3.2). Such sampling could perhaps also be performed for weight. However, here we adopt a simpler approach whereby each missing weight value is imputed once via linear interpolation between the two nearest observed values for that vole. The robustness of inference to other sensible imputed weight values obtained by using a growth model is investigated in Section 4.3.

### 2.5. Details of the Markov models for individual diseases

The remainder of this section gives a brief description of each disease in the study and describes the HMM that is used to model it. All transition probabilities are time dependent since some of the covariates are time dependent; however, for ease of notation we drop any explicit reference to this time dependence. A more detailed description of the host resources that are required by these parasites and a discussion about host immune responses can be found in Telfer *et al.* (2008).

#### 2.5.1. *Bartonella* species

*Bartonella* is a genus of bacteria that infects mammals (including humans), which is usually transmitted by arthropods. The species that are investigated here are transmitted by fleas (Bown *et al.*, 2004). We assume that the effect of other diseases and covariates on the probability that a vole will recover from a particular *Bartonella* species after the second (third, fourth etc.) lunar month is the same as for the effect on the probability of recovery after the first month; there are no grounds for assuming otherwise. However, since the majority of *Bartonella* infections last for 1 month and only a few last more than 2 (Birtles *et al.*, 2001; Telfer *et al.*, 2008) the overall probabilities of recovering after the first and second month are likely to be different. Additionally a vole’s chance of contracting a particular *Bartonella* species for the first time might be different from the chance of contracting it again after recovery from it in the past, although, again, there is no reason to assume that the effects of other diseases and covariates on this are likely to be different. This suggests that each *Bartonella* species could be sensibly modelled by using a Markov chain with four states: 1, no infection; 2, new infection; 3, old infection; 4, uninfected but has had a past infection. However, the observed sequence indicates either negative N or positive P status. In particular, an observation of 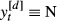 corresponds to a hidden process of 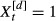 or 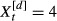 with likelihood vector 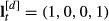, and an observation of 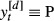 corresponds to 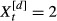 or 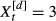 with 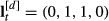. The time inhomogeneous transition probability matrix from time *t* to time *t*+1 for this Markov chain is 
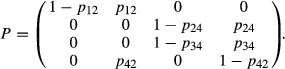
(2)

Each transition probability is governed by a logistic regression as follows: 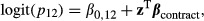
(3)

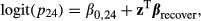
(4)

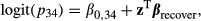
(5)

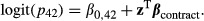
(6)

As justified above, we use the same vector of covariate effects 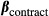 for the two probabilities related to contracting the particular *Bartonella* species. Similarly we use the same covariate effects for the two probabilities relating to recovery from the disease, 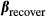; we allow only the intercepts to differ. This assumption prevents a further increase in the, already large, number of parameters to be estimated. For example, the logistic model for the probability of contracting *B. taylorii* for the first time at lunar month Lm+1 will be 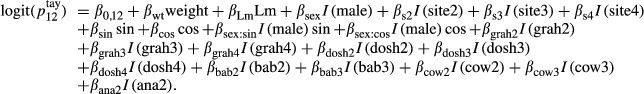


Here and elsewhere *I*(·) denotes the indicator function, and [disease]*x* is a statement that the hidden chain for [disease] is in state *x*.

#### 2.5.2. Babesia

*Babesia microti* can cause haemolytic anaemia in infected hosts. It is a chronic infection, which is to say that once a host has been infected it is never again free of the disease. The effect of a *Babesia* infection on the probabilities of contracting or recovering from one of the other diseases may depend on whether the *Babesia* infection is acute (in its first month) or chronic.

We therefore model *Babesia* by using a Markov chain with the following three states: 1, no infection; 2, acute infection; 3, chronic infection. Here the likelihood vector that connects the states with the observations is analogous to that for *Bartonella* species but ignoring state 4. The transition matrix is 
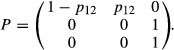


As in the previous section, a logistic regression relates 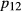 to the covariates, including the states of the other diseases.

#### 2.5.3. Anaplasma

*Anaplasma phagocylefthilum* is a tick-borne bacterium that causes the disease granulocytic ehrlichiosis in humans. In the data set there are relatively few positive records for *Anaplasma* and thus little power to ascertain transition probabilities and covariate effects from a third state of, for example, ‘currently uninfected but was previously infected’. Therefore, we use a two-state Markov chain with transition probability matrix 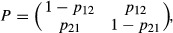 and separate logistic regressions relating 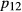 and 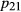 to the covariates, including the states of the other diseases. This therefore is the only disease for which the underlying Markov model is not hidden.

#### 2.5.4. Cowpox

In voles and other wild rodents, infection with cowpox virus is known to last for approximately 4 weeks (Bennett *et al.*, 1997). The diagnostic test, however, detects antibodies to the virus, not the virus itself. Antibodies appear approximately 2 weeks after contracting the infection but then remain present in the bloodstream of a vole for the rest of its life (Bennett *et al.*, 1997). Since the disease lasts for approximately 1 month we model the progression as a Markov chain with three states: 1, antibodies absent and disease absent; 2, antibodies present and disease present; 3, antibodies present and disease absent. Therefore, the form of the transition matrix and the relationship between the states and the response is identical to that for *Babesia*. The difference is in the interpretation: here state 3 corresponds to a positive response but absence of the disease, whereas for *Babesia* state 3 corresponds to a positive response which means that the disease is present.

## 3. Bayesian approach

### 3.1. Choice of prior

#### 3.1.1. Initial probability distribution

The likelihood (Section 2.2) and the forward–backward algorithm (Section 2.3) require, for each disease, the initial distribution 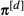 of the Markov chain on the set of states for that disease at the first observation time for each vole. Our time inhomogeneous Markov chains admit no limiting distribution and so the popular choice of setting the initial distribution to the limiting distribution of the chain is not available to us.

We choose then to estimate this distribution for each disease through our Gibbs sampler in Section 3.2. We choose independent and relatively vague Dirichlet priors 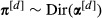, with 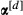 set to a vector of 1s with length equal to the cardinality of state space 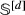. This is equivalent to a uniform prior on each 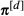.

#### 3.1.2. Prior distributions for the regression parameters

A similar longitudinal data set to the one that we analyse was also available to us. This additional data set arises from an earlier, 3-year study which was conducted using the same sampling design, but where the response for *Bartonella* was a single indicator for presence and absence, rather than an indicator for each species. For each of *Babesia,Anaplasma* and cowpox we could therefore fit a logistic regression to a subset of the additional data set as briefly described in Sections 1.1 and 2.1, except that the three indicator covariates for presence or absence of each *Bartonella* species were replaced with a single indicator covariate for presence or absence of at least one *Bartonella* species. Parameter estimates from these analyses were used to inform our choice of prior for similar parameters in our main analysis.

Since the additional data set does not distinguish the *Bartonella* species, there is not an exact correspondence between parameters from the simple analyses and the parameters in our main model, and some of the parameters in our main model have no counterpart in the simple analyses. The priors for each 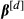 (*d*=1,…,*D*) in our main analysis are independent and Gaussian with covariance matrices that we denote by 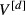. For *Babesia,Anaplasma* and cowpox, where parameters do approximately correspond, we set the prior mean for the parameter in our main analysis to the maximum likelihood estimate for the corresponding parameter in the simple analysis of the additional data set and the block of 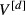 that is associated with these parameters to nine times the analogous block from the simple analysis. Where no corresponding maximum likelihood estimate exists, the prior mean was set equal to 0 and the block of 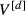 was a diagonal matrix where the diagonal elements were set to 9.

### 3.2. Adaptive Metropolis-within-Gibbs algorithm

In our data set, the target parameter ***β*** can be naturally partitioned into six subblocks: one for each disease. In the Gibbs scheme we wish to update the covariate parameters for each given disease *d*, 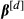, by using a *random-walk Metropolis* (RWM) step (see for example Gilks *et al.* (1996)); however, the efficiency of a given RWM algorithm depends heavily on the choice of the variance of the proposal jump, 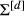. Both Roberts and Rosenthal (2001) and Sherlock and Roberts (2009) suggested that an RWM algorithm might achieve near optimal efficiency when 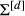 correctly represents the general shape of the target distribution, e.g. if it is proportional to the variance of 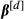 or the inverse curvature at the mode. We therefore generalize the *adaptive RWM* algorithm that was described in Sherlock *et al.* (2010) to an *adaptive Metropolis-within-Gibbs* algorithm on *D* subblocks.

Let 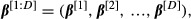, where 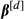 is the parameter set for the *d*th subblock. A single iteration starts from an initial value 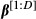, cycles through all the subblocks updating each in turn and finishes with 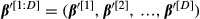. In the update for the *d*th block the current and proposed values are respectively 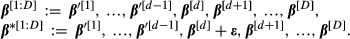
(7)

Here the proposal jump ***ɛ*** in expression (7) for the *d*th subblock at the *n*th iteration is sampled from a mixture distribution: 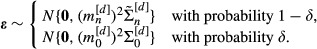
(8)

Here *δ* is a small positive constant, which we set to 0.1, 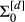 is a fixed covariance matrix and 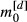 is set to the theoretically derived value 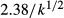 (Roberts and Rosenthal, 2001), where *k* is the dimension of 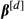. The matrix 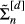 is the estimated variance of 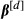 by using the sample from the Markov chain to date. The scaling factor 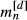 for the adaptive part is initialized to 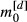. Sherlock *et al.* (2010) detailed the adaptation of the scaling factor at each iteration, which leads to an equilibrium acceptance rate of 25%, which is close to the optimal acceptance rate (approximately 23%) that was derived by Roberts and Rosenthal (2001).

The conditional likelihood for 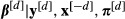 is calculated by using the forward part of the forward–backward algorithm for disease *d*. This provides the conditional posterior for 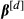 and hence the acceptance probability for the proposed value of 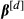. The states of the hidden Markov chain for disease *d* can then be sampled from their conditional distribution given the observed data for that disease, 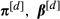, and the states of the other diseases using the backward part of the forward–backward algorithm.

Given the states of the hidden chain for a particular disease *d* and, in particular, the initial state for each vole, the conditional conjugacy of the Dirichlet distribution allows straightforward sampling from the conditional posterior for 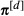.

We therefore simulate from the joint posterior distribution of the coefficients of the logistic regressions for the transition probabilities, the hidden disease states and the initial probability distribution of the hidden states with the following MCMC algorithm.

At the start of the current iteration of the chain let the covariate parameters be 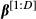, let the hidden states be 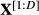 and the initial distribution of the hidden states be 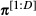; denote their values at the start of the next iteration as 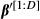, 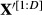 and 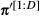 respectively.

Each iteration of the Gibbs sampler is as follows.

*Step 1*: perform an adaptive RWM update according to 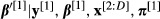.
*Step 2*: simulate the hidden states for the first disease from 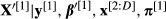.
*Step 3*: simulate the initial probability distribution of the chain for the first disease 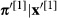.
*Step 4*: perform an adaptive RWM update according to 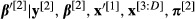.
*Step 5*: simulate the hidden states for the second disease from 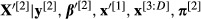.
*Step 6*: simulate 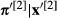.
  ⋮
*Step* 3*D*−2: perform an adaptive RWM update according to 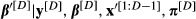.
*Step* 3*D*−1: simulate 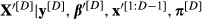.
*Step* 3*D*: simulate 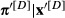.

The adaptive RWM step requires the fixed covariance matrix 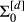. For each disease, a separate non-adaptive RWM step was performed for the logistic regressions coefficients that are associated with the HMM for this disease that are not associated with the other diseases, e.g. weight and sex. The block of 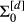 that is associated with these covariates was estimated directly from this run. Each of the remaining *β*-coefficients was given a small proposal variance and was assumed to be uncorrelated with any of the other coefficients. Also to ensure a sensible non-singular 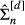, for each disease, proposals from the adaptive part were only allowed once at least 1000 proposed jumps had been accepted.

## 4. Analysis and results

### 4.1. Convergence of the algorithm and model diagnostics

All the computationally intensive parts of the algorithm were coded in C within an R (R Development Core Team, 2012) wrapper. On a computer with an Intel Nehalem 2.26 GHz central processor unit, 100000 iterations of the algorithm took approximately 3 h.

**Figure 2:**
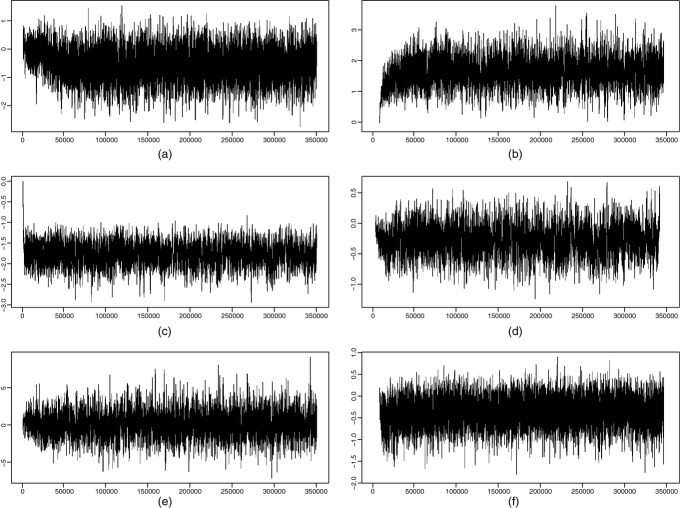
Trace plots of the *β*-coefficients for (a) cow12.tay2, (b) tay24.bab2, (c) dosh12.bab3, (d) grah24.tay4, (e) ana21.grah3 and (f) bab12.tay3

Three independent Markov chains of length 350000 were generated from the algorithm in Section 3.2; each chain was started from a different position. Six of the 233 trace plots from one of the chains are reproduced in Fig. 2. For most of these, over the first few tens of thousands of the iterations the variance of the proposal increases as the adaptive algorithm learns the shape of the posterior; this was so in many of the 233 trace plots.

**Figure 3:**
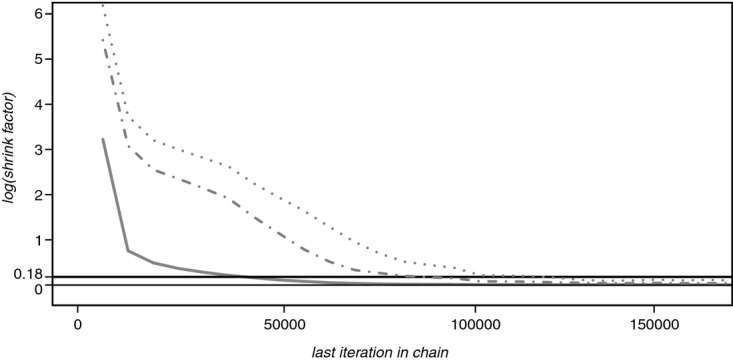
Combined Gelman–Rubin statistics for all 233 *β*s, 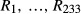 (the statistics are plotted on a log-scale against iteration number, along with the ideal ratio of log(1) (0) and the threshold of log(1.2) (0.18) suggested in Gelman (1996)): 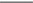, mean Gelman–Rubin point estimator (1/233)
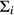

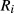
; 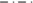, maximum Gelman–Rubin point estimator 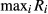; 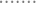, maximum 97.5% quantile estimate 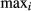

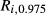

The Gelman–Rubin statistic (Gelman and Rubin, 1992) *R* was calculated from the three chains for each of the 233 components of ***β***. Fig. 3 shows the mean of the estimated *R*-statistics, the maximum of the estimated *R*-statistics along with the maximum of the 97.5% quantiles of *R*-statistics, plotted against iteration number. The plot suggests that a burn-in period of 150000 iterations should be more than sufficient. Inference is therefore performed using the final 200 000 iterations from each of the three runs combined.

To assess model fit we examine the posterior predictive distribution of the data (see Robert *et al.* (1999)). We chose, at random, 100 captures where all six diseases were observed and created an alternative data set where all diseases for these captures were marked as missing. We refitted the model and estimated the posterior probabilities 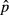 that these artificially removed observations are positive. A Hosmer–Lemeshow test (e.g. Collett (2003)) for each disease provides a *p*-value for the null hypothesis that each of the true (binary) observations arises from a Bernoulli trial with the given posterior probability. For the six diseases we obtained *p*-values of 0.443 (*B. doshiae*), 0.188 (*B. grahamii*), 0.061 (*B. taylorii*), 0.141 (*Babesia*), 0.56 (*Anaplasma*) and 0.322 (cowpox).

### 4.2. Posterior inference

We are interested in interactions between diseases, e.g. in whether or not the presence or absence of disease 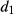 affects the probability of a change of state for disease 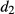. In each logistic regression for each transition matrix, we therefore examine the coefficients that correspond to the states of the other diseases. We are also interested (for *Bartonella*) in whether or not the status of an infection (new or old) affects the chance of recovery, and in whether or not a previous infection affects the chance of a new infection with the same species; these correspond respectively to the contrasts 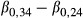 and 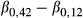.

Formal model choice, e.g. via reversible jump MCMC sampling (Green, 1995), is computationally infeasible here. Instead we take a high posterior probability that a given parameter or contrast is positive (or a high probability that it is negative) as indicating a potentially important effect. For an individual parameter we might consider *P*(positive)>0.975 or *P*(positive)<0.025 as indicating a likely effect. We are, however, interested in a total of 116 parameters and contrasts, which raises a similar problem to that of multiple testing in classical statistics. Although considering probabilities below 0.025 or above 0.975 to indicate a possible interaction, we therefore take probabilities below 0.000 25 and above 0.999 75 as indicating a very probable interaction.

Table 4 shows those parameters for which the posterior probability of positivity is either above 0.975 or below 0.025. Table 4 shows the posterior median, a 95% credibility interval and the posterior probability that the parameter is positive.

**Table 4:** Posterior summaries of model parameters of interest for which the posterior probability of positivity is either above 0.975 or below 0.025†

*Transition probability*	*Covariate*	*Median*	*95% credibility interval*	*Posterior probability*
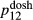	tay2	−1.0124	(−1.8708, −0.1554)	0.0111
	tay3	−1.1077	(−2.0584, −0.1877)	0.0096
	tay4	−1.0304	(−1.8046, −0.2278)	0.0077
	bab2	−1.5636	(−2.4247, −0.8039)	0.0001
	bab3	−1.7702	(−2.3095, −1.3235)	0.0000
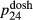	bab2	2.9613	(1.6459, 4.5298)	1.0000
	bab3	3.3386	(1.775, 5.5524)	1.0000
	cow2	−1.01	(−2.0937, −0.0691)	0.0171
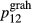	bab2	−1.2579	(−2.0506, −0.5881)	0.0000
	bab3	−2.0294	(−2.6841, −1.5096)	0.0000
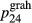	bab2	2.7818	(1.1981, 5.0649)	0.9999
	bab3	1.7288	(0.8899, 2.754)	1.0000
	ana2	1.0284	(0.0116, 2.1709)	0.9764
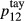	bab2	−1.6933	(−2.6563, −0.815)	0.0001
	bab3	−1.3346	(−1.8338, −0.8754)	0.0000
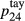	bab2	1.5824	(0.702, 2.5404)	0.9997
	bab3	1.7513	(1.0015, 2.6279)	1.0000
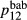	cow3	−0.4939	(−0.9799, −0.0206)	0.0200
*Contrasts*				
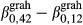		−1.3248	(−5.6452, −0.2774)	0.0076
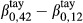		−4.472	(−8.6604, −2.1214)	0.0015
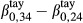		1.1762	(0.555, 1.8601)	0.9998

† For each parameter the posterior median, a 95% credibility interval and the probability that the parameter is greater than 0 is provided. Each parameter arises from a logistic regression coefficient for a particular transition probability in the HMM for a particular disease. The disease and transition appear in the first column, and the second column indicates the particular disease and state that is influencing the transition probability. State 1 is always taken to be the baseline. The contrasts 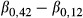 and 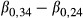 are defined in Section 4.2.

Firstly, and most clearly, the presence of *Babesia* decreases the probability of contracting *Bartonella* and increases the probability of recovery from *Bartonella*. This is true for both chronic and acute *Babesia* infections and for all three species of *Bartonella*. There is no evidence for the reverse interaction, i.e. for the presence of *Bartonella* affecting the chance of contracting *Babesia*.

For two of the three *Bartonella* species (*B. taylorii* and *B. grahamii*) it appears that a vole is less likely to be reinfected following previous exposure whereas it is more likely to recover from an old infection of *B. taylorii* than a new one. Furthermore, there seems to be a decrease in the probability of contracting *B. doshiae* when a vole has been exposed to *B. taylorii* whether or not it is still infected. Finally, infection with *Anaplasma* appears to increase the probability of recovery from *B. grahamii*; there was perhaps some evidence for the same interaction with *B. doshiae* with posterior probability 0.9593. There is no evidence of a change to the probability of recovery from *B. taylorii* (probability 0.416). It is also possible that a current infection with cowpox virus hinders recovery from *B. doshiae* and previous exposure to cowpox prevents infection with *Babesia*.

### 4.3. Sensitivity analysis

Three somewhat arbitrary choices were made in the set-up of our model and priors: the interpolation scheme that fills in missing weight values, the prior for the initial distribution for the state of the HMM for each disease and vole, and the exact relationship between parameters that are estimated in the simple analysis of the alternative data set and priors for parameters in the HMMs for the main data set.

An alternative for each of these choices is described below. For each alternative three further chains of length 350000 were created and checked for convergence. Then any sizable changes in the conclusions that would be drawn from the posterior distributions of the parameters were noted.

In the main analysis, missing weight values were filled in via linear interpolation. As an alternative we considered the logistic growth curve which was proposed in Burthe *et al.* (2009). We assumed Gaussian residuals for the logarithm of weight and allowed the logistic growth parameters to depend on covariates such as the sex of the vole and the time of year; some of the coefficients were also allowed to include subject-specific random effects. More details are provided in Xifara (2012).

The initial distributions for the states of the HMMs for the diseases are assigned independent Dirichlet priors with the parameter for each disease, 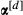, a vector of 1s. As an alternative prior we set each 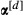 to be a vector of 2s.

In the main analysis, where there was a rough correspondence between a parameter in the simple analysis of the alternative data set and a parameter in the main analysis, we centred the Gaussian prior in the main analysis on the maximum likelihood estimate from the simple analysis and set the covariance matrix to be nine times the estimated covariance matrix from the simple analysis (Section 3.1.2). As an alternative we use vague but proper Gaussian priors for all parameters.

Parameter estimates with the alternative weight scheme or with the alternative prior distribution of the hidden states were very similar to the estimates from the main set-up. For all the significant covariates none of the posterior probabilities changed by more than 0.005. However, the use of vague priors for the parameters noticeably affected one of the 21 covariates in Table 4. The effect on a vole’s probability of contracting *Babesia* when the vole had been exposed to cowpox became apparently unimportant, with posterior probability changing from 0.02 to 0.093. No additional covariates became potentially important (i.e. *p*<0.025 or *p*>0.975) in any of the three alternative runs.

## 5. Discussion

We have described a coupled discrete time HMM for interactions between diseases in a host and used it to analyse data from a longitudinal study of field voles with records of six different pathogens. The Markov model offers a more detailed description than the existing modelling approach that is described in Section 1.1. Furthermore, by explicitly dealing with the missing observations (which comprise approximately 50% of the data set), the inference methodology that we introduce can use more of the data than the existing standard inference methodology.

Inference is performed via a Metropolis-within-Gibbs sampler that cycles through the diseases and, for each disease conditional on the hidden states of all of the other diseases, samples from the parameters of the logistic regressions for the transition probabilities of the HMM using an adaptive RWM step and then from the exact distribution of the hidden states given these parameters. These two steps use respectively the forward and backward parts of the forward–backward algorithm.

The forward–backward Gibbs sampler (e.g. Chib (1996), Scott (2002) and Fearnhead and Sherlock (2006)) also uses the forward–backward algorithm; however, the motivation is different. The forward–backward Gibbs sampler does not use the likelihood from the forward recursion directly, as this would require a Metropolis–Hasting update; instead the backward recursion provides a sample from the posterior distribution of the hidden states given the parameters. Owing to the conditional conjugacy structure of the problems that is targeted by the forward–backward Gibbs sampler it is then possible to sample exactly from the conditional posterior for the parameters given the hidden states and thus to avoid the Metropolis–Hastings step and the associated tuning. Our logistic regression model for the transition probabilities does not allow a simple Gibbs step for updating the parameters conditionally on the hidden states, and so we content ourselves with a Metropolis–Hastings step for the parameters and, for efficiency of mixing, do not condition on the hidden states for the current disease. After the Metropolis–Hastings step we *then* sample from the hidden states for the disease so that these can be used as covariates for the other diseases; in effect, we therefore sample from the joint conditional distribution of the parameters and the hidden states for the disease. The forward–backward algorithm could be avoided entirely by updating the logistic regression parameters conditionally on the hidden states for *all* diseases, and by sampling from the distribution of each individual hidden state conditionally on all the other hidden states and the transition parameters (e.g. Robert *et al.* (1993) and Robert and Titterington (1998)). However, we believe that the correlation between hidden states and between these states and the parameters would have led to a very poorly mixing MCMC chain; Scott (2002) has discussed the first aspect of this.

Brand (1997), Saul and Jordan (1999), Rezek *et al.* (2000), Zhong and Ghosh (2002) and Natarajan and Nevatia (2007) all examined inference for coupled HMMs. The directed acyclic graph for the HMMs that they considered is the same as in Fig. 1; however, to allow recursions that are similar to those in the forward–backward algorithm all—except Rezek *et al.* (2000)—made the simplifying assumption that the transition probability for a given chain conditional on the others is separable: 
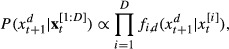
 for some collection of non-negative functions 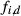. Moreover the computational complexity increases with the square of the sum of the number of states in each chain and, in practice, each of Brand (1997), Saul and Jordan (1999), Rezek *et al.* (2000), Zhong and Ghosh (2002) and Natarajan and Nevatia (2007) considered only two chains. We wish to apply logistic regression rather than to assume separability and to consider six chains; furthermore computational complexity of our algorithm increases with the sum of the squares of the number of states in each chain. Rezek *et al.* (2000) performed Bayesian inference via a Gibbs sampling algorithm that is qualitatively similar to our own; the fully conjugate structure that is used does not, however, allow for the effects of any covariates.

We now examine the most major findings of Section 4.2 and briefly discuss the biological insights that they offer. For *B. taylorii*, voles are more likely to recover from an old infection than a new one, which is to be expected given that more complete histories for individuals indicate that most infections last for only 1 month. Previous data also indicate that infections by *B. doshiae* may last longer than infections by *B. taylorii* and *B. grahamii* (Telfer *et al.*, 2007). Also, for *B. grahamii* and *B. taylorii*, previous infection by the species appears to grant some form of immunity to that species, suggesting that hosts can develop an effective acquired immune response. To date, there has been conflicting evidence for such a response in wild populations, suggesting that immune responses may vary between host species and/or *Bartonella* species (Birtles *et al.*, 2001; Kosoy *et al.*, 2004; Bai *et al.*, 2011). Interestingly also infection by *B. taylorii* appears to provide immune cross-protection to infection by *B. doshiae*.

We found, for voles currently infected with *Babesia*, both a reduction in susceptibility to *Bartonella* and an increase in the probability of recovery from *Bartonella* over the next lunar month. We also found no evidence that a current *Bartonella* infection might influence susceptibility to *Babesia* over the next month. Telfer *et al.* (2010) found that the *Babesia* covariates, both at time 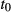 and at time 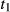, were significant for predicting the probability of catching *Bartonella* between 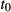 and 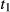. The *Bartonella* covariate at time 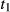 is also found to be significant in predicting susceptibility to *Babesia*, apparently contradicting our findings. However, as mentioned in Section 2.1, Telfer *et al.* (2010) allowed both the current (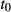) and future (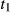) state of each disease covariate to influence the probability that, for the disease that is being treated as a response, a vole tests positively at time 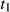 given that it tests negatively at time 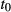. An assumption of only the negative effects of *Babesia* on *Bartonella* infections that is apparent from inference for our HMM, and no other dependence between *Bartonella* and *Babesia*, is sufficient to lead to a negative correlation between *Bartonella* and *Babesia* at any given time. Consider two groups of voles: those with *Babesia* at 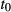 (group A) and those without *Babesia* at 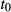 (group B). Since *Babesia* is a chronic infection, group A voles are more likely to have *Babesia* at 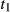 than are voles from group B. However, since *Babesia* impacts negatively on the probability that a vole has *Bartonella*, group A voles are less likely to have *Bartonella* at 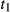 than voles from group B. Since the effect of *Babesia* on *Bartonella* is so pronounced (Table 4), it is certainly believable that this negative correlation could be sufficiently strong that each of the two diseases at 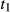 appears as an important covariate for the other.

In the application which we have considered, missingness was believed to be independent of disease state; in other scenarios, such as those considered in Pradel (2005), the probability that a given subject will be observed might depend on the states of each of the HMMs. This could be accommodated within our methodology through a further logistic regression for the probability of being observed given the set of hidden states and other covariate information, and several other minor changes as detailed in Pradel (2005).
